# Young Adults’ Knowledge and Attitudes Regarding “Music” and “Loud Music” Across Countries: Applications of Social Representations Theory

**DOI:** 10.3389/fpsyg.2019.01390

**Published:** 2019-06-25

**Authors:** Vinaya Manchaiah, Fei Zhao, Pierre Ratinaud

**Affiliations:** ^1^Department of Speech and Hearing Sciences, Lamar University, Beaumont, TX, United States; ^2^Audiology India, Mysore, India; ^3^Department of Speech and Hearing, School of Allied Health Sciences, Manipal Academy of Higher Education, Manipal, India; ^4^Centre for Speech Language Therapy and Hearing Science, Cardiff Metropolitan University, Cardiff, United Kingdom; ^5^Department of Hearing and Speech Science, Xinhua College, Sun Yat-sen University, Guangzhou, China; ^6^LERASS Laboratory, University of Toulouse, Toulouse, France

**Keywords:** hearing loss, loud music, music listening, recreational noise exposure, attitude, social representation

## Abstract

Exposure to loud music, especially by young people, has significantly increased in recent years as a result of (a) advancements in technology in terms of personal music players and smart mobile phones, and (b) streaming of music through these devices. The World Health Organization (WHO) estimates that some 1.1 billion teenagers and young adults are at risk of developing hearing loss due to exposure to recreational noise such as music. It is suggested that knowledge and attitude of young adults toward music has bearing upon their music listening habits and thereby influences who is at risk of developing music induced hearing loss. Hence, researchers from various fields have tried to understand the knowledge and attitude of young adults regarding loud music. However, there is some criticism of attitude studies as there is little relation between expressed attitude and behavior. Some recent studies have explored the social representations of music and loud music using the Social Representations Theory (SRT). It has been suggested that social representation is more fundamental than attitude (or in other words social representation informs attitude), hence, it has a better relation to behavior. The current paper: (1) provides an overview of studies on knowledge and attitude of young adults toward loud music, (2) discusses the limitations of attitude theories and introduces SRT, and (3) provides a summary of social representation studies on “music” and “loud music” in young adults from different countries.

## Introduction

Music refers to the “art of combining sounds or sequences of notes into harmonious patterns pleasing to the ear and satisfying to the emotions; melody” (Sheriff, 2014, p. 1). Over the last decade, there has been a substantial increase in the number of people listening to music, and also in the amount of time people spend listening to music (Fobers, 2017). This increase can be attributed to easy access to music as a result of: (a) advancements in technology in terms of personal music players and smart mobile phones, and (b) streaming of music through these devices. It is noteworthy that younger adults listen to music significantly more than middle-aged and older adults (IPSOS Connect, 2016). Moreover, in the last decade, music listening has become a significant public health hazard, especially in adolescents and young adults. Listening to loud music can result in various hearing disorders (Zhao et al., 2010; Jiang et al., 2016). The World Health Organization (WHO) estimates that some 1.1 billion teenagers and young adults are at risk of developing hearing loss due to exposure to recreational noise such as music (World Health Organization [WHO], 2015).

The effects of loud music are related to both the intensity and duration of exposure. For example, listening to music slightly over moderate levels (i.e., 80–85 dB A) for longer durations (i.e., over 8–10 h a day) can result in various hearing disorders (NIOSH, 1998). Although people are wary of adverse effects of loud music listening, music is generally considered a positive aspect of life in most cultures. Hence, it is suggested that knowledge and attitude of young adults toward music has bearing toward their music listening habits, and thereby influences who is at risk of developing music induced hearing loss (MIHL; Zhao et al., 2011). Moreover, understanding the perception of young adults from different cultural backgrounds may help develop strategies for hearing health promotion specific to individuals from different cultural backgrounds (Zhao et al., 2011, 2015).

Researchers from various fields have tried to understand the relation between knowledge and attitude of young adults regarding loud music and the risk of developing hearing disorders (e.g., Widén and Erlandsson, 2004; Widén et al., 2006; Vogel et al., 2007; Landälv et al., 2013). However, there is some criticism of attitude studies focusing on music listening as there is little relation between expressed attitudes and behavior in relation to listening to music (for review see Zhao et al., 2011). Some recent studies have explored the social representations of “music” and “loud music” using the Social Representations Theory (SRT) (Manchaiah et al., 2017a, b, 2018). It is argued that social representation informs attitudes; hence, it has better relation to behavior (Moliner and Tafani, 1997). For this reason, studying music and loud music perception using the SRT may be fruitful when compared to other theoretical frameworks (i.e., health behavior change theories focusing on attitude).

The current paper: (1) provides an overview of studies on knowledge and attitude of young adults toward loud music; (2) discusses the limitations of attitude theories and introduces SRT; and (3) provides a summary of social representation studies on “music” and “loud music” in young adults from different countries.

## Knowledge and Attitude of Young Adults Toward Loud Music

Knowledge is defined as understanding a subject or information, which is acquired through experience or education by perceiving, discovering, or learning (Zack, 1999). Attitude is regarded as a psychological phenomenon, not only referring to people’s insight experience, but also includes people’s behavior tendency toward “like or dislike” (Albarracin et al., 2005). It is formed through a process of self-evaluation influenced by various factors (such as cognitive, affective, motivational, and behavioral components). Understanding the knowledge and attitude of young adults toward loud music provides essential information for developing effective hearing health education programs in order to raise awareness, increase knowledge, improve attitude, and consequent change (Zhao et al., 2011).

Various studies have examined the knowledge and attitude toward loud music exposure, and also the association between knowledge and attitude toward habits of music, listening, and behaviors for protective listening in adolescents and young adults. Lass et al. (1987) conducted a survey showing that students had very poor knowledge in certain areas related to hearing health. Although the majority of participants were aware of the risk for hearing damage caused by noise exposure, limited knowledge was found in the areas related to the mechanisms of hearing loss and hearing health awareness, particularly regarding the facts of permanent irreversible hearing damage caused as a result of loud music exposure. Nearly fifty percentage of participants (i.e., 48.5%) thought that noise induced hearing loss (NIHL) could be treated by medical intervention. A similar survey by Chung et al. (2005) was conducted using an internet-based survey, which asked adolescents and young adults questions related to general health (e.g., sexually transmitted diseases, depression, drug and alcohol use, smoking, nutrition, weight issues, and acne), as compared to any concerns of hearing health. The results derived from this study showed that hearing health was the least concern when compared with various general health matters. Study participants’ poor knowledge is evidenced by only 9% of participants having hearing health education at school, and only 16% were aware of causes of hearing loss by reading, listening or watching materials related to hearing loss. Furthermore, a recent study by Hunter (2018) also showed that poor knowledge of hearing symptoms and related damage led to a lack of concern for hearing health. The author adapted a qualitative approach to investigate knowledge and attitudes toward loud music and subsequent behaviors in young adults, such as the use of hearing protection. Five over-arching themes were derived from this study, which include: (1) enjoying loud music; (2) previous hearing damage; (3) peer behaviors and opinions; (4) lack of knowledge and concern; and (5) hearing not being a priority. Of these, lack of knowledge and concern was mainly expressed by participants as, “unsure about the risk of loud music level and exposure duration”, and “misunderstanding the implications and mechanisms underlying the hearing loss”. In addition, the participants did not consider hearing problems a priority, and as a result, they were reluctant to compromise their lifestyle or change their listening behaviors toward the enjoyment of loud music. As indicated in the study by Hunter (2018), lack of appropriate levels of knowledge and understanding resulted in the lack of hearing health concerns. In this study, participants (i.e., young adults) believe that loud music enhances their experience of leisure activities and enjoyment. Moreover, they feel it creates a positive impact upon their mood, clearly indicating the association between knowledge and attitude (Hunter, 2018).

The associations between knowledge and attitude have been explored in previous studies. For example, Chung et al. (2005) revealed an important change in attitude associated with increased knowledge of in hearing health. Gallagher (1989) reported that approximately 15–20% more students were willing accepted to use ear protection at a concert after they were informed and educated about the dangers of loud music. In addition, Landälv et al. (2013) explored adolescents’ attitudes toward loud music associated with self-perceived auditory symptoms and a number of psychological variables (e.g., norms, preparedness to take risks, and risk-judgment in noisy situations). They found that self-perceived auditory symptoms (e.g., hearing sensitivity change, permanent tinnitus) were related to less tolerant attitudes toward loud music. This result is consistent with the findings obtained from the study by Widén et al. (2009). Their findings also imply that the attitude toward loud music exposure was significantly associated with self-experienced hearing symptoms, but not to changes measuring hearing sensitivity. In the study by Hunter (2018), some participants expressed negative attitudes they had with previous hearing symptoms. However, such negative attitudes did not necessarily lead them to take actions (such as using hearing protectors or withdrawing from noisy situations), unless they started experiencing more severe or permanent hearing problems. There is still a stigma of using hearing protection devices due to peer opinions and behaviors. Landälv et al. (2013) pointed out that variables such as social norms, preparedness to take risks, and risk-judgment were associated with attitudes toward loud music. This finding is consistent with the study by Widén (2013), who suggests that social norms play an important role in influencing attitudes and subsequent behavioral changes. Overall, although there is mixed evidence about the relation between knowledge and attitude toward loud music in young adults, the general consensuses based on literature reviews is that poor attitude is likely due to not understanding the seriousness of hearing health, as the consequences of loud music exposure are not immediate. Hence, hearing health education programs may not offer much in terms of raising awareness in the young and in developing specific knowledge. However, raised awareness of consequences does not, in itself, change behavior (Zhao et al., 2011).

Some important influencing factors related to attitudes toward loud music exposure and the use of hearing protection have been identified (Widén and Erlandsson, 2004), for example, demographic factors (i.e., age, gender, educational level, ethnicity), music preference, physical activity, socioeconomic factors, and cultural perspectives. Of those, the cultural factor has considerable influence upon listening behavior, and thus it is crucial to consider this factor in determining an effective health listening education program. A previous study by Widén et al. (2006) compared cultural differences in attitudes toward loud music and the use of hearing protection between American and Swedish young adults. Their study showed that the attitudes were more “positive” toward loud music in the American sample as compared to the Swedish sample. Moreover, the use of hearing protection was much lower within the American sample as compared to the Swedish (Widén et al., 2006). Their explanation for the attitude differences between these two western countries is attributed primarily to the increased awareness of harmful effects caused by loud music, and an acceptance of earplugs in Sweden. Because campaigns regarding the dangers of environmental noise and loud music have been launched regularly in Sweden, significant and beneficial changes have occurred in protective listening habits and behaviors.

Therefore, the views and opinions toward loud music exposure obtained from young adults in different countries may reflect real-life experiences associated with their listening culture, which will help identify influential ways to raise awareness and disseminate hearing health education. Better understanding of the knowledge, attitudes, and listening behaviors influenced by psycho-social factors provides important information to help in further development of effective guidance for intervention, and recommendations for appropriate policies and strategies of hearing health education programs for adolescents and young adults. Moreover, there is also a need to explore different theoretical approaches in order to better understand the issues related to loud music listening in young adults.

## Limitations of Attitude Theories and Introduction to Social Representations Theory

### Limitations of Attitude Theories

The concept of “attitude” has been the focus of social psychology since the emergence of the field (Allport, 1935; Albarracin and Shavitt, 2018). An attitude is an evaluation of an object or thought. An attitude object can be about anything that a person holds in mind, which can range from the mundane to the abstract, focusing on things, people, groups, or ideas. However, the definition of attitude inherently involves much diversity. Bohner and Dickel (2011) suggest that “*attitude definitions characterize attitudes as either constructed on the spot from accessible information or as stable entities that are stored in memory. The two types of definition draw different lines of evidence to account for attitudes’ context sensitivity versus stability over time*” (p. 411). The concept of attitude has been applied to many disciplines, including marketing (e.g., attitudes toward products and services), advertising (e.g., attitudes toward promotional activities and advertisements), political behavior (e.g., attitudes toward political candidates, parties, or voting), and health (e.g., attitudes toward protective behaviors, new medications, or the health system).

Several health behavior change theories have been used to explain attitudes and behaviors of individuals toward music listening behavior (Sobel and Meikle, 2008; Manchaiah, 2012). For example, Rawool and Colligon-Wayne (2008) studied the auditory lifestyle and beliefs of college students toward exposure to loud sounds using the Health Belief Model (HBM). In another study, Sobel and Meikle (2008) examined applications of health behavior theories to hearing conservation interventions. Studies using health behavior change theories in relation to attitudes toward loud music can be summarized into three aspects, which include: (a) the use of interpersonal theories in predicting the relation between knowledge, attitude, and beliefs, which may help in developing hearing conservation education programs; (b) applications of transtheoretical stages-of-change models to evaluate the individual’s readiness for change; and (c) the use of HBM in promoting positive hearing health behavior (for review see Manchaiah, 2012). These theories generally focus on attitude and involve a number of important issues. First, empirical correlations between attitude and behaviors are weak, which may suggest that attitude and behaviors do not reflect the same dimension (Andrich and Styles, 1998). Second, it has been suggested that attitude theories take the individual perspective, and that they lack social perspective (Farr, 1994; Howarth, 2006; Bidjari, 2011; Marková, 2017). Researchers have attempted to broadenor stretch, the concept of “attitude” to allow for a better understanding of the social aspects. For example, the Theory of Reasoned Action takes account of social norms and social norms can be seen as a version of social representations (Fishbein and Ajzen, 1975). However, others may disagree with such a claim, suggesting that the concept “attitude” does not adequately incorporate the social aspects to any significant degree. Hence, it has been argued that a truly *social* psychology needs new conceptual tools to address the social attitude (Howarth, 2006). The SRT is one alternative that allows consideration of “societal aspects” as its prime focus.

### Introduction to Social Representations Theory

The SRT focuses on our everyday knowledge and beliefs about the world, which involves built-in social interaction with others. Social representation can be viewed as a system of values, ideas and practices (Moscovici, 1961). The orientation in the physical world can be facilitated by such a system. Also, the system of values, ideas and practices provides a code for naming and classifying various aspects of each individual’s respective world, enabling communication (Moscovici, 1973). From this perspective, representations can be seen as expressions of our modern culture or collective historical beliefs that people hold about phenomena found in their respective environments (Chaib and Orfali, 1995). In other words, social representation can be seen as common knowledge that is collectively elaborated upon by groups in intercommunication processes. In practice, representations guide our communication and behavior, and create a certain approach to the world (Jodelet, 1989). In summary, it is noteworthy that the SRT has been developed to understand and explain how a particular group as a whole perceive various phenomena. Hence, attitude is recognized as a result of representation of populations, suggesting that representations are more fundamental than attitudes. Various researchers have used the SRT to describe and understand social phenomena (Marková et al., 2000; Morant, 2006; Linton et al., 2013; Manchaiah et al., 2015a).

Some researchers have taken a historical perspective to examine the similarities and differences between attitude and social representations. Farr (1994) highlighted that there is some similarity between Moscovici’s notion of social representations and Thomas’s notion of social attitudes (Thomas and Znaniecki, 1918-1920), although more current studies on attitude are no longer social as previously designated. According to Jaspars and Fraser (1984) there is some similarity between social representations and social attitudes, but also many differences between social attitudes and attitudes in general. Also, Howarth (2006) argued that attitudes are conceptualized from an individual perceptive, whereas social representations are conceptualized from the social perspective.

The SRT helps overcome some of the fundamental limitations raised about attitude theories. First, while the studies based on “attitude” provide important information about various social phenomena, a poor correlation between the attitude and the actual behavior is also noted in empirical research (Kollmuss and Agyeman, 2002). However, studies based on SRT reveal collective perceptions of members of groups or communities about a phenomenon and the influence of such perceptions upon actual behavior (Jodelet, 1989; Wagner and Hayes, 2005). Second, according to the World Health Organization’s – International Classification of Functioning, Disability, and Health (ICF), the environmental factors, in particular “societal attitudes, norms, practices and ideologies,” may have an important influence on how individuals manage their health and well-being (World Health Organization [WHO], 2001). However, attitude research does not consider broader environmental factors limiting its theoretical reach. On the other hand, the social aspects are fundamental to which SRT of populations, In addition, some researchers have argued that the studies based on attitude are problematic due to the measurement scales used (see Jaspars and Fraser, 1984; Marková, 2017). In most studies, attitude is measured using the Likert scale, and researchers tend to develop the questions that are deemed important enough to be examined. However, “free association task,” which is one of the more popular methods of gathering data in studies using the SRT, tends to have an open approach in eliciting aspects that are important to respondents. For these theoretical and practical reasons, SRT can be more fruitful in studying various phenomena including music listening habits and behavior.

## Summary of Studies on Social Representation of “Music” and “Loud Music” in Young Adults

In our recent cross-cultural exploratory research project, we examined the social representation of “music” (Manchaiah et al., 2017a) and “loud music” (Manchaiah et al., 2017b, 2018) in young adults (aged 18–25 years) from India, Iran, Portugal, the United Kingdom, and the United States. In this section, we provide a summary of these already published studies (Manchaiah et al., 2017a, b, 2018).

### Study Method

The study sample included 534 young adults (mean age = 21.04 years, SD = 2.5 years) from India (*n* = 110, mean age = 21.05 years, SD = 2.2 years), Iran (*n* = 100, mean age = 22.24 years, SD = 2.6 years), Portugal (*n* = 101, mean age = 19.72 years, SD = 1.8 years), United Kingdom (*n* = 122, mean age = 22.02 years, SD = 2.6 years), and United States (*n* = 101, mean age = 19.99 years, SD = 1.8 years). Of these, 43.4% of the participants were males. 27.5, 62.2, and 10.3% of the participants had compulsory, secondary, and tertiary education, respectively. The mean listening hours of the participants per week was 14.47(SD 19.7) h.

A cross-sectional survey design was used in this study. The data was collected using a convenience sampling method by approaching young adults at universities and city center shopping malls. Generally, the surveys were conducted during the middle of the day and evenings, although we did not keep strict time-logs for the data collection. The data was collected in the cities of Mysore (Karnataka, India), Terhan (Iran), Porto (Portugal), Cambridge (United Kingdom), and Beaumont (United States). These cities are considered medium to large sized cities, suggesting that the data was mainly coming from urban society.

The study participants were asked to report four to five words or phrases that immediately came to their minds when they thought about “music” and “loud music.” Following this, they were asked to consider each word or phrase reported, and to indicate if that word or phrase carried positive, neutral or negative connotations. Finally, they were asked to provide some demographic information. This method of data collection is called “free association task,” which is a common method used to study the semantic universe of social representation (Abric, 1994). The original questionnaire was in English, which was used in the United Kingdom and the United States. However, a translated version using a well-accepted forward–backward translation method (Beaton et al., 2000) was used in India (Kannada), Iran (Farsi), and Portugal (Portuguese).

At first, the individuals’ open responses were categorized using the qualitative content analysis (Graneheim and Lundman, 2004). In the next step, the data were subject to a series of quantitative analyses using the IRaMuTeQ software.^1^ These included: (a) Chi-square test to examine the response distributions of connotations; (b) similarities analysis to examine the frequencies and also inter-relations between different categories reported (Flament, 1965); and (c) cluster analysis to identify groups of individuals with similar characteristics based on patterns in reported responses.

### Summary of Studies

Table 1 presents the response categories (based on qualitative content analysis), frequency of responses to each category, and also the number of participants reporting at least one response for each category. As indicated in Table 1, the participants’ responses fell into 18 main categories for music (Manchaiah et al., 2017a) and 19 main categories for loud music (Manchaiah et al., 2017b). “Positive emotions and actions” was the single most frequently occurring category for music, whereas the categories “Negative emotions and actions” and “Positive emotions and actions” were the two most frequently occurring categories for loud music. Also, the majority of participants reported at least one response toward “Positive emotions and actions” (i.e., 88%) and “Negative emotions and actions” (i.e., 67%) for music and loud music, respectively. While the categories shared numerous commonalities, some of the categories were unique to music and loud music. For example, the categories “Memories,” “Positive quality of life,” and “Religion and spirituality” were only reported for music. However, categories “Ear and hearing problems,” “Hearing protection,” “Physical ailment,” and “Public Awareness” were only reported for loud music.

**TABLE 1:** Percentage of categories reported in different countries and the percentage of respondents mentioning individual categories.

**No.**	**Categories**	**Music**		**Loud music**	
			**% respondents**		**% respondents**
			**mentioning**		****mentioning****
		**% responses**	**this category**	**% responses**	**this category**
1	Acoustics (e.g., sound, decibel, noise, loudness, intensity)	4.1	16.7	8.0	34
2	Body Structure (e.g., ear, vocal cords)	0.5	2.4	0.9	4
3	Ear and hearing problems (e.g., hearing loss, tinnitus, otalgia)	–	–	8.9	36
4	Entertainment (e.g., MTV, radio)	2.5	11.2	0.3	2
5	Form of escape (e.g., freedom, distraction, isolation, dream)	2.5	11.1	1.6	7
6	Friends and family (e.g., neighbors, friends, family)	1.5	6.6	1.9	9
7	Hearing protection	–	–	0.3	1
8	Location (e.g., festivals, work, concerts, bar)	2.5	11.1	7.6	29
9	Memories (e.g., moments and nostalgia)	1.4	6.2	–	–
10	Music genre (e.g., disco, jazz, rock, heavy metal)	3	14.2	3.6	14
11	Music terminology (e.g., rhythm, melody, music, song)	8.8	26.8	1.2	6
12	Musical artists, groups, or bands (e.g., specific artist’s name, band)	4	14.6	1.1	4
13	Musical instruments (e.g., piano, flute, guitar)	4.4	16.1	0.8	3
14	Nature (e.g., sea, mountains, rain)	1.1	4.7	1.1	6
15	Negative emotions or actions (e.g., sadness, discomfort, displeasure, confusion, irritation)	3.3	13.7	28.2	67
16	Party and alcohol (e.g., nightlife, DJ, drunkeness)	1.9	8.1	5.4	24
17	Personal listening devices and transducers (e.g., earphones, phones, mp3, speakers)	2	7.3	3.6	16
18	Physical ailment (e.g., pain, sick, headache …)	–	–	7.3	31
19	Positive emotions or actions (e.g., joy, happiness, singing, dancing, fun)	55.8	88	17.1	47
20	Positive quality of life (e.g., wellness, well-being, life quality)	0.6	3	–	–
21	Public awareness (e.g., being aware of adverse effects of loud music)	–	–	1.1	5
22	Religion and spirituality (e.g., spirit, God)	0.9	3.8	–	–

**FIGURE 1:**
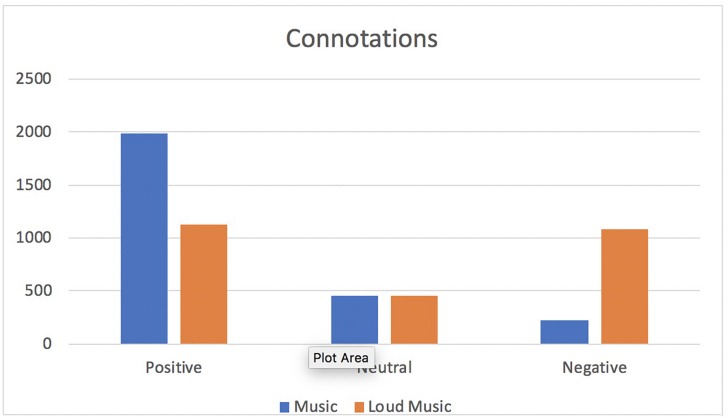
Positive, neutral, and negative connotations related to “music” and “loud music.”

Figure 1 shows the positive, neutral or negative connotations associated with each response for music and loud music. For music, the responses included 74.7, 16.9, and 8.4% of positive, neutral and negative connotations. For loud music, the responses included 42.5, 17, and 40.5% of positive, neutral and negative connotations. Chi square analysis (with a 3 × 2 cross tab for connotations vs. music/loud music) indicated a significant association between connotations and music or loud music (Chi square = 800.73, *p* < 0.00001).

**FIGURE 2:**
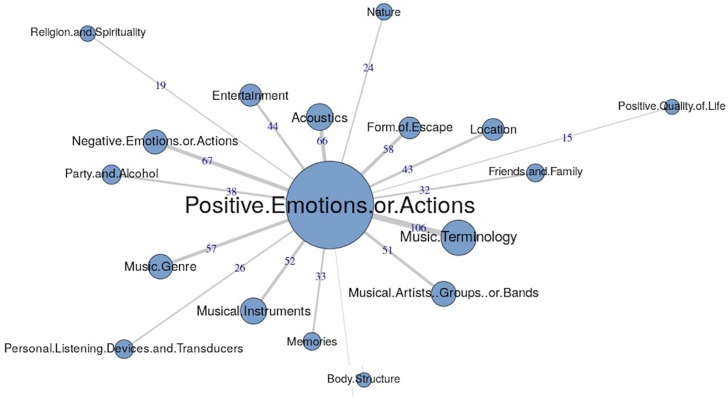
Global maximum tree showing main categories related to music listening and their associations with each other (*n* = 534).

**FIGURE 3:**
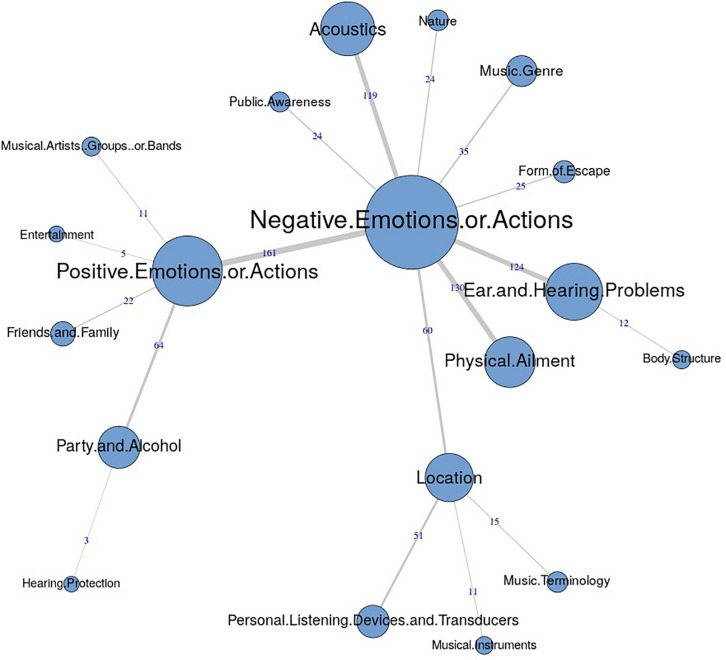
Global maximum tree showing main categories related to loud music listening and their associations with each other (*n* = 534).

**FIGURE 4:**
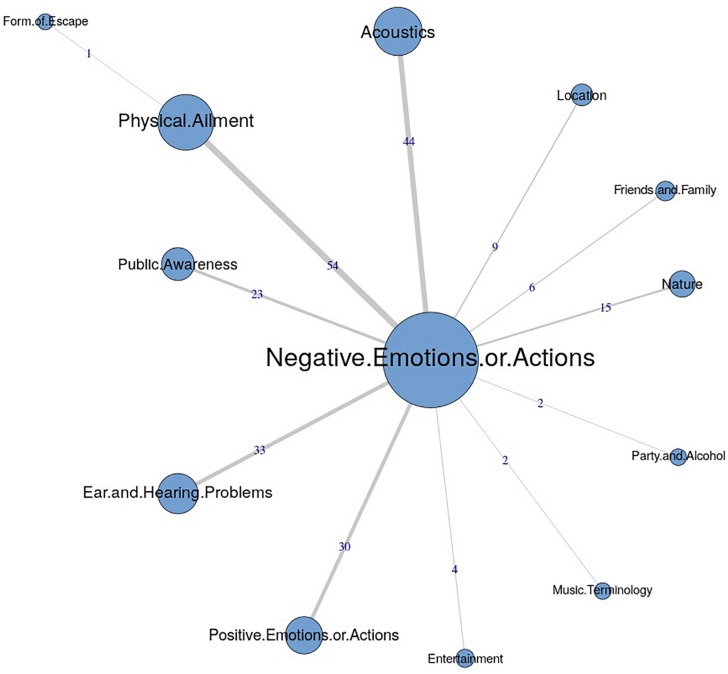
Maximum tree for India showing main categories related to loud music listening and their associations with each other (*n* = 110).

**FIGURE 5:**
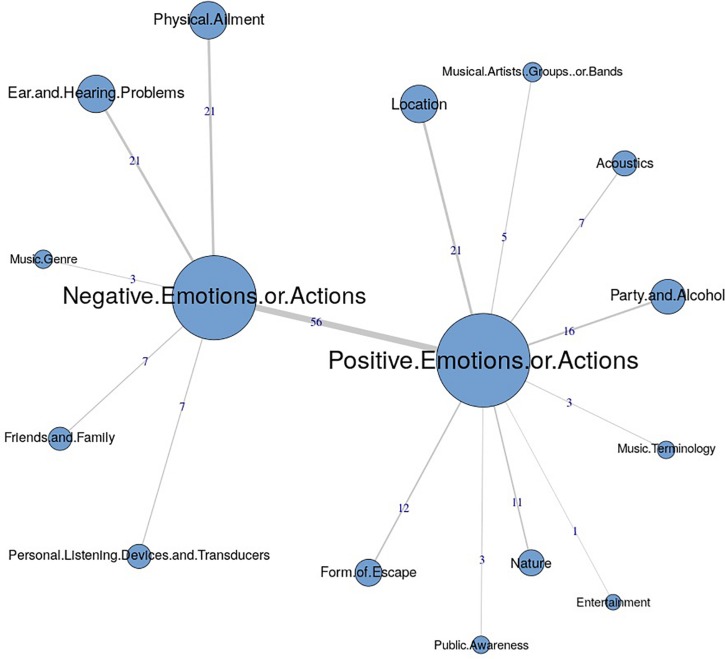
Maximum tree for Iran showing main categories related to loud music listening and their associations with each other (*n* = 100).

**FIGURE 6:**
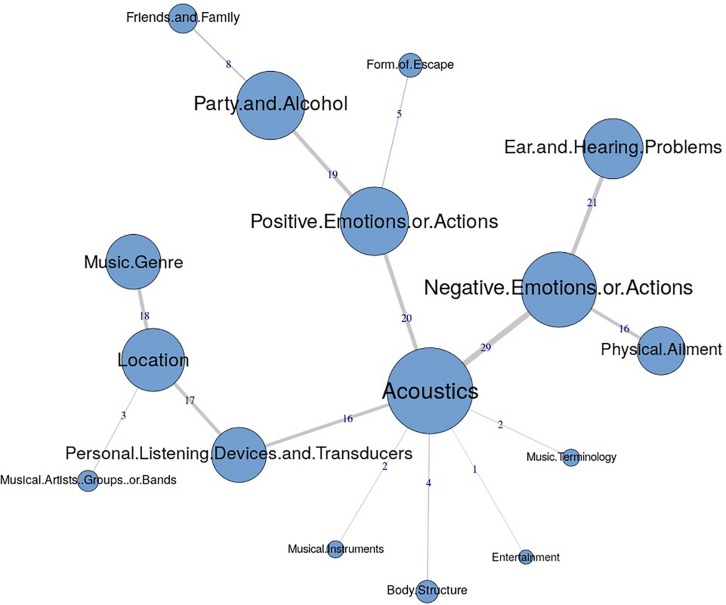
Maximum tree for Portugal showing main categories related to loud music listening and their associations with each other (*n* = 101).

**FIGURE 7:**
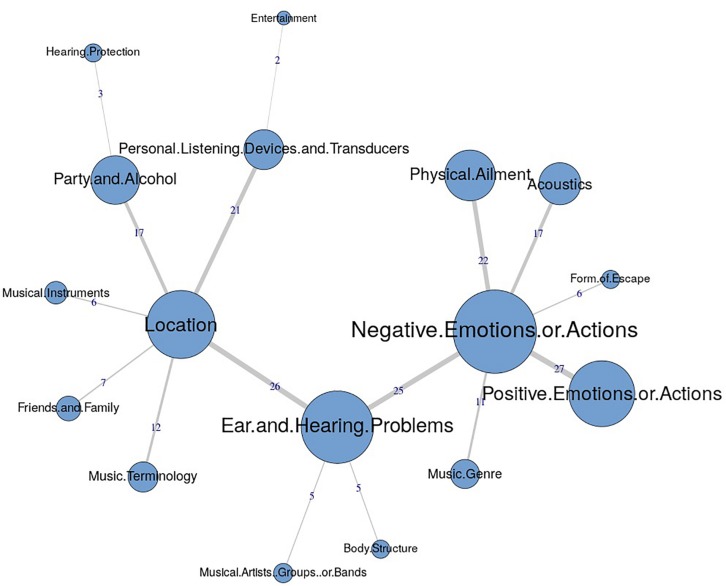
Maximum tree for United Kingdom showing main categories related to loud music listening and their associations with each other (*n* = 122).

**FIGURE 8:**
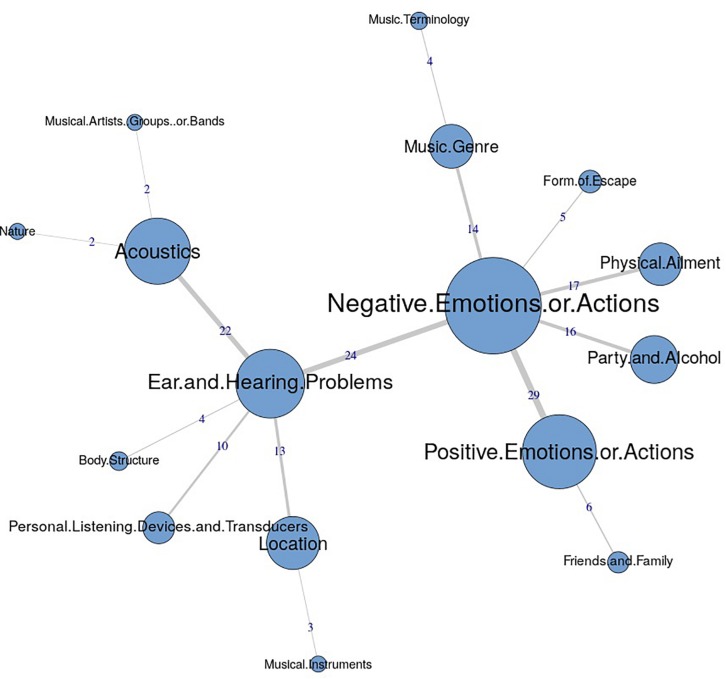
Maximum tree for United States showing main categories related to loud music listening and their associations with each other (*n* = 101).

The similarities analysis results are presented as maximum tree indices (see Figures 2–8). Examination of these results provides insights into the common categories reported in each country, and also demonstrates the interrelation between response categories. In these figures, the nodes represent the frequency of each category, and the line connecting the nodes represents interrelation between categories. Theoretically, any number of connections (i.e., lines) can be possible between the categories. However, a threshold is set to only highlight the connections that appear to be stronger, indicating the relative importance of these connections. Examination of similarities analyses of music data reveals minimal differences between countries (i.e., India, Iran, Portugal, the United Kingdom, and the United States), as the “positive emotions and actions” was the single most frequently reported category in all countries (see Manchaiah et al., 2017a). Hence, we report the global maximum tree (see Figure 2), which indicates that category “Positive emotions and actions” forms the single most noticeable node for music perception, and all of the other categories are interrelated and connected to this category. On the contrary, the global maximum tree for loud music (see Figure 3) suggests that the category “Negative emotions and actions” was the most frequently occurring category, followed by the “Positive emotions and actions” category (Manchaiah et al., 2017b). However, examination of maximum tree (see Figures 3–7) for loud music indicates substantial cross country (or cross-cultural) differences among countries (Manchaiah et al., 2017b). “Negative emotions and actions” was the single most frequently occurring category in India (see Table 1). “Negative emotions and actions” and “Positive emotions and actions” were the two most frequently occurring categories in Iran (see Figure 5). The similarities analysis results in Portugal, the United Kingdom and the United States were more deviant from India and Iran, indicating various categories including “negative emotions and actions,” “positive emotions and actions,” “acoustics,” “party and alcohol,” and “ear and hearing problems” (see Figures 6–8). It is interesting to note that respondents from these western countries were able to associate “Ear and hearing problems” with loud music, when compared to respondents from India and Iran. These differences may be related to the events and activities associated with loud music in those countries or cultures. For instance, in India, loud music is often associated with celebratory events such as weddings, festivals and religious events. Also, in Iran, loud music is associated with religious activities. Hence, participants from those countries may not associate loud music with aspects such as “Ear and hearing problems.” However, it is surprising to see that in India, the largest response category regarding loud music was “Negative emotions and actions,” although as suggested earlier, the loud music relates to celebratory events in India. Based on the anecdotal reports, we speculate that this may be a result of: (a) poor sound quality due to less advanced technology used, producing distorted and uncomfortable sounds; and (b) preferences of the Indian population to have music at more moderate levels.

The cluster analysis of responses (18 categories) to music did not reveal any interesting results (unpublished data). This may be a result of “Positive emotions and actions” being the single-most frequently occurring category of responses (i.e., 55.8%) related to music. This means perception about music did not differ much between participants. However, cluster analysis of responses (19 categories) to loud music resulted in four clusters (see Table 2). These included: (a) emotional oriented perception (included 29.7% of respondents); (b) problem-oriented perception (included 20.5% of respondents); (c) music and enjoyment-oriented perception (included 37.7% of respondents); and (d) relaxation-oriented perception (included 12.9% of respondents) (Manchaiah et al., 2018). Table 2 shows the categories that were significantly more common in these clusters. Chi square analysis showed that participants from India are more likely to be in cluster 1 and 2, participants from Portugal and the United Kingdom are more likely to be in cluster 3, and participants from Iran are more likely to be in cluster 4 (Manchaiah et al., 2018). No statistical significance was observed between participants from the United States and clusters, suggesting that they were distributed across the clusters and are not likely to be in any of the clusters. These preliminary results from the exploratory studies indicate that loud music can create different perceptions among different groups of individuals. Also, there are cross-cultural differences involving the perception of loud music.

**TABLE 2:** Cluster analysis of “loud music” social representation.

**Cluster (%)**	**Main variables**
Cluster 1:	Negative emotions and actions
Emotional oriented perception (29.7%)	Positive emotions and actions
	Physical aliment
Cluster 2:	Ear and hearing problems
Problem oriented perception (20.5%)	Physical aliment
	Negative emotions and actions
Cluster 3:	Location
Music and enjoyment-oriented perception (37.7%)	Personal listening devices and transducers
	Music genre
	Party and alcohol
	Family and friends
	Musical instruments
	Body structure
	Music terminology
	Music artists, groups, or bands
	Entertainment
Cluster 4:	Nature
Relaxation oriented perception (12.9%)	Form of escape
	Public awareness
	Positive emotions or actions

## General Discussion

The current manuscript provides an overview about the studies on knowledge and attitude of young adults toward loud music. In particular, recent studies on social representation of “music” and “loud music” in young adults from different countries provide some interesting and novel insights (Manchaiah et al., 2017a, 2017b, 2018). First, it is clear that social representations toward “music” and “loud music” are markedly different. “Music” is generally considered to elicit positive emotions and actions, whereas “loud music” is considered to elicit both positive and negative emotions, as well as actions and a range of other issues related to music (i.e., music genre, location), social events and activities (e.g., party and alcohol) and health (e.g., ear and hearing problems). Second, the studies highlight that social representations toward “music” seem to be universal showing commonalities across countries, whereas social representations toward “loud music” seem to be markedly different across countries. However, some caution is necessary to interpret these results as other factors may have influenced these results. For instance, the minimal differences noted in response to music may be related to urbanization of the study sample, although we cannot confirm or deny this based on current study results. Moreover, the social representation studies discussed in this manuscript treat individuals from different countries as individuals from different cultures, although there is much more diversity within these countries. Hence, the concept of culture can be defined more precisely based on a system of beliefs, values, and practices. Nonetheless, these findings strengthen the idea that understanding “why do people like loud sounds?” and “how these preferences change across individuals and more importantly social groups?” may be important in developing appropriate health promotion strategies (Welch and Fremaux, 2017).

The ideologies and culture of any given society may also affect the form and style of the music people prefer. For instance, individuals’ preferences in music is likely to be dependent on the different dimensions of social stratification (Feld, 1984; Dolan and Sharot, 2011), which to some degree can influence people’s music-related behavior, including listening habits. In the recent review about attitude and attitude change published in the Annual Review of Psychology, Albarracin and Shavitt (2018) highlighted that “*attitude theorizing, as developed in the West, offers an incomplete account of how attitudes function and are structured in non-Western cultures, where normative processes play a stronger role in shaping attitudes and their functions*” (p. 230). Moreover, they suggest that “*attitudes must be studied within social networks and in relation to historic and other environmental events*” (p. 321). This can be achieved with the use of SRT as societal aspects are central to this theory. The studies on social representations of “music” and “loud music” have identified the sociocultural issues associated with these objects. Therefore, music experience, music perception, and music listening behavior should be considered within a sociocultural context, associated with its own history, invention, identity, and belonging.

Moreover, it has been suggested that social representations are generally stable and would require a strong external influence in order to create any given change (Flament, 1987). However, it has not been established if the attitudes and also social representations of young adults toward loud music is stable. This is due to not only the population (e.g., young adults) but also to the object of representation (e.g., music, loud music) which is highlighted in the current paper. For instance, Albarracin and Shavitt (2018) suggest that “*attitudes of the Millennial generation differ from those of prior generations in being more conservative fiscally and politically, liberal socially (e.g., support for egalitarian gender roles and same-sex marriage), individualistic, self-focused, and materialistic*” (p. 320). Furthermore, aspects such as media seem to focus on music on a regular basis which may form and re-form the attitude and social representations about this object, which in turn may influence individuals and societal behavior in terms of music listening. While these arguments about unstable representations may question the validity of existing studies in an ongoing re-formation of attitude and social representations, it also highlights the opportunity to influence social representations of loud music in a positive manner. For example, it has been suggested that the media can be a strong influence which can help change social representations. Hence, future studies should examine the influence of the media (both news media and social media) upon the knowledge, attitude, and social representations of young adults regarding “music” and “loud music.” Moreover, studies can also focus on developing optimal strategies to influence the policies and public health directives addressing loud music exposure, particularly with the use of the media.

Finally, while we highlight some of the limitations of attitude theories in this manuscript, we still argue that there is value in studies using attitude theories, although there is room for new theoretical approaches such as SRT. For instance, we have highlighted the criticism about attitude theories for the issues surrounding the measurement of attitudes based on self-report Likert scales. However, in recent years attitude research has been marked by more frequent use of implicit, response-time-based measures (De Houwer et al., 2009; Greenwald et al., 2009; Bohner and Dickel, 2011). We are not aware of any studies which have applied such an approach to understanding music listening behavior in young adults. Hence, we believe that future attitude studies in this area should employ more current methodologies. Moreover, there is a great need for using new theoretical approaches such as SRT in better understanding dynamic issues such as music listening habits in young adults. However, the social representations of music and loud music discussed in this manuscript are based on exploratory studies. Hence, caution should be taken while interpreting and generalizing these results. Nevertheless, we argue that there is a need for triangulation in terms of both the methodological and the theoretical approaches we use in studying the knowledge, attitude, and behavior of music listening in young adults.

## Author Contributions

VM developed the concept and prepared the first draft of the manuscript. FZ and PR provided the comments and edits of the manuscript.

## Conflict of Interest Statement

The authors declare that the research was conducted in the absence of any commercial or financial relationships that could be construed as a potential conflict of interest.

## Footnotes

1 http://www.iramuteq.org/
